# Classification and neuroimaging of ependymal tumors

**DOI:** 10.3389/fped.2023.1181211

**Published:** 2023-05-23

**Authors:** Weiya Mu, Hisham Dahmoush

**Affiliations:** ^1^ Department of Radiology, Stanford Health Care, Stanford, CA, United States; ^2^ Department of Radiology, Lucile Packard Children’s Hospital, Stanford, CA, United States

**Keywords:** ependymal tumor, ependymoma, subependymoma, myxopapillary ependymoma, spinal ependymoma, ependymoma subgroups

## Abstract

Ependymal tumors arise from the ependymal cell remnants of the cerebral ventricles, the central canal of the spinal cord, or the filum terminale or conus medullaris, although most pediatric supratentorial ependymomas do not exhibit clear communication or abutment of the ventricles. In this article, we discuss the classification, imaging characteristics, and clinical settings of these tumors. The WHO 2021 classification system has categorized ependymal tumors based on histopathologic and molecular features and location, in which they are grouped as supratentorial, posterior fossa (PF), and spinal. The supratentorial tumors are defined by either the ZFTA (formerly RELA) fusion or the YAP1 fusion. Posterior fossa tumors are divided into group A and group B based on methylation. On imaging, supratentorial and infratentorial ependymomas may arise from the ventricles and commonly contain calcifications and cystic components, with variable hemorrhage and heterogeneous enhancement. Spinal ependymomas are defined by MYCN amplification. These tumors are less commonly calcified and may present with the “cap sign,” with T2 hypointensity due to hemosiderin deposition. Myxopapillary ependymoma and subependymoma remain tumor subtypes, with no change related to molecular classification as this does not provide additional clinical utility. Myxopapillary ependymomas are intradural and extramedullary tumors at the filum terminale and/or conus medullaris and may also present the cap sign. Subependymomas are homogeneous when small and may be heterogeneous and contain calcifications when larger. These tumors typically do not demonstrate enhancement. Clinical presentation and prognosis vary depending on tumor location and type. Knowledge of the updated WHO classification of the central nervous system in conjunction with imaging features is critical for accurate diagnosis and treatment.

## Introduction

Ependymal tumors are glial neoplasms that arise from the ependymal remnants of the cerebral ventricles, the central canal of the spinal cord, and the filum terminale or conus medullaris. In the pediatric population, these tumors account for approximately 5% of primary brain tumors and 22% of the spinal cord and cauda equina tumors (1).

Significant changes have been made between the 2016 and the WHO 2021 classifications of central nervous system tumors (Table 1) (2, 3). The WHO 2021 classification system has recatergorized ependymal tumors based on histopathologic and molecular features and location (4). New categories of supratentorial ependymomas include ZFTA fusion-positive and YAP1 fusion-positive. Posterior fossa (PF) ependymomas are divided into group A (PFA) and group B (PFB) based on the methylation group. In spinal ependymomas, there is a new group defined by MYCN amplification, which indicates a more aggressive tumor. There is no change in the classification of myxopapillary ependymoma and subependymoma. At this time, tumors with molecular markers are not assigned a WHO grade, given the limited research on these subtypes, although most ependymal tumors are grade 2 or 3 (4).

**Table 1:** Summary of the 2021 WHO classification of tumors of the central nervous system. WHO grades are not assigned to the new molecular groups added in the latest version. Myxopapillary ependymoma is upgraded from grade 1 to grade 2.

Location	Molecular grouping	WHO grade
Supratentorial	ZFTA fusion-positive	
	YAP1 fusion-positive	
	Supratentorial ependymoma	2, 3
Posterior fossa	Posterior fossa group A	
	Posterior fossa group B	
	Posterior fossa ependymoma	2, 3
Spinal	MYCN amplified	
	Spinal ependymoma	2, 3
Spine	Myxopapillary ependymoma	2
Brain	Subependymoma	1

There are, of course, tumors that do not fit the previously mentioned diagnostic criteria. For tumors for which molecular analysis has not been obtained or has technically failed, the suffix “not otherwise specified” (NOS) is used. The suffix “not elsewhere classified” (NES) is used when the necessary diagnostic tests have been successfully performed, but the clinical, histologic, immunohistochemical, and genetic features do not match an existing WHO category (5).

In this article, we discuss the classification, clinical settings, and imaging characteristics of these tumors. Given the importance of imaging in the diagnosis of these tumors, it is important to correlate imaging findings with the molecular groups. We also discuss the differential considerations for these tumors.

## Supratentorial ependymomas

Supratentorial ependymomas are predominantly seen in children and adolescents. Patients with these tumors typically present with headaches, seizures, and focal neurologic deficits. In adults, these tumors generally arise from the ventricular margins, whereas in pediatric patients they often do not have clear communication or abutment of the ventricles. There are two molecular markers defined in the WHO 2021 classification, namely, ZFTA fusion and YAP1 fusion, as discussed below. These markers are more commonly found in pediatric supratentorial ependymomas, with a median age of 8 years for ZFTA fusion–positive tumors and 1.4 years for YAP1 fusion–positive tumors (6).

The ZFTA fusion–positive supratentorial ependymomas were previously categorized as RELA fusion. While the ZFTA or C11orf95 gene often fuses with the RELA gene as the primary oncogenic event, it can also fuse with other partner genes. These tumors have worse survival outcomes compared to YAP-1 fusion supratentorial ependymomas, with a 10-year overall survival of approximately 50% (6).

**Figure 1:**
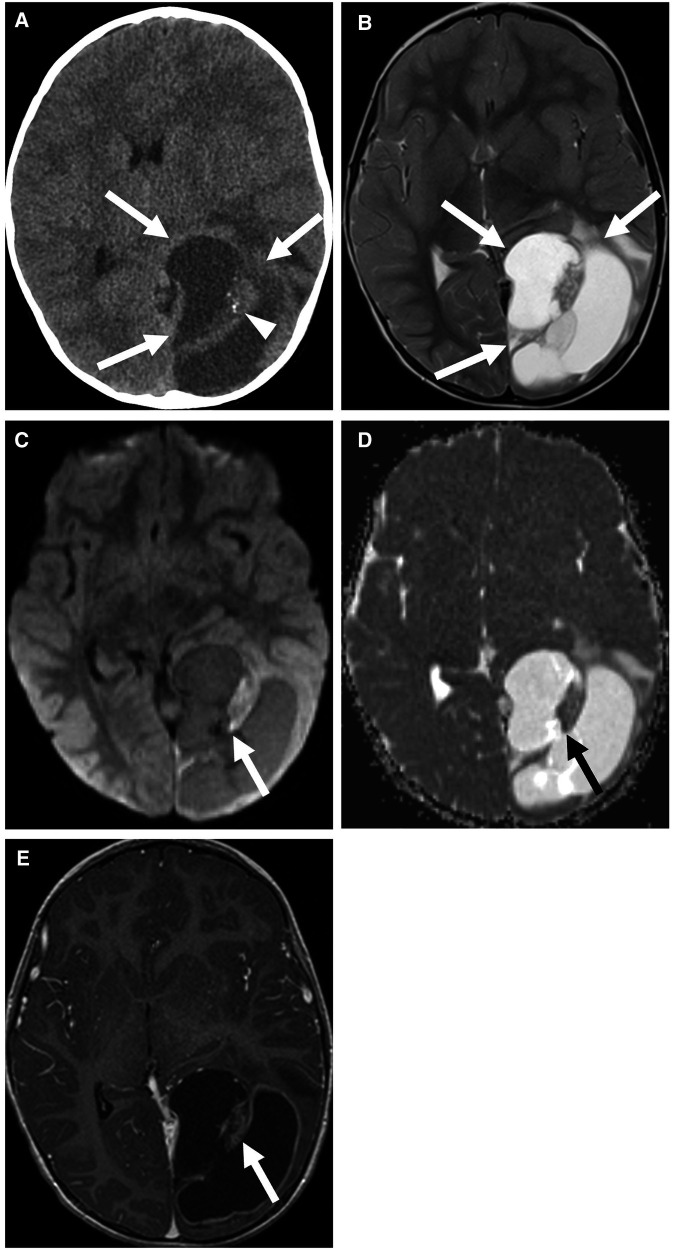
Supratentorial ependymoma, ZFTA fusion-positive in a 3-year-old boy. Non-contrast CT (**A**) and axial T2 (**B**) images show a cystic and solid mass centered in the left occipital lobe (arrows) with calcification of the solid component (arrowhead). The solid component of the mass shows mild diffusion restriction (**C,D**) and enhancement (**E**) (arrows). There is also an associated mild rightward shift of the midline.

These tumors tend to be solid and cystic masses, with heterogeneous enhancement and restricted diffusion of the solid component (Figure 1) (7). Necrosis, calcification, and hemorrhage are common. Distinguishing these tumors from other masses based on imaging alone is difficult.

YAP1 fusion–positive tumors are less common than ZFTA fusion–positive tumors, accounting for approximately 7% of supratentorial ependymomas (6). Despite the large size at presentation, these tumors have a better prognosis than the ZFTA fusion supratentorial ependymomas, with an overall survival period of 10 years at rates ranging between 88% and 100% (8). There is a slight prevalence in female patients.

**Figure 2:**
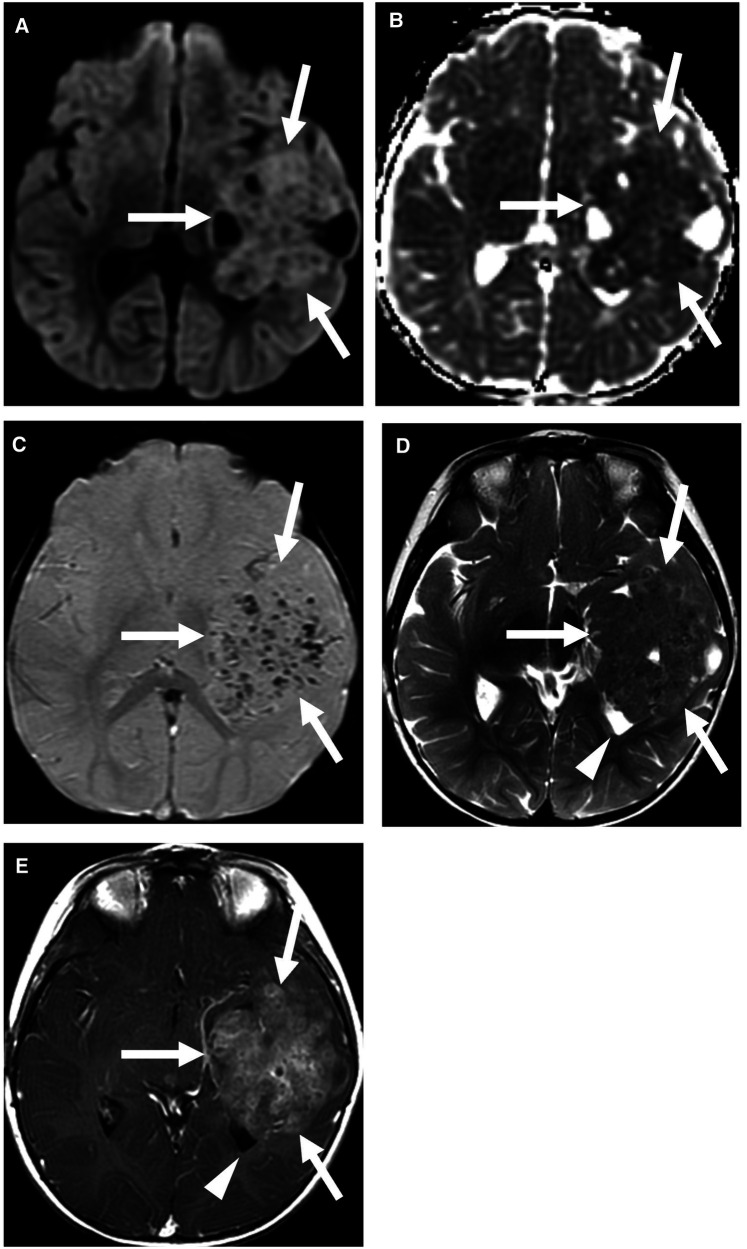
Supratentorial ependymoma, YAP1 fusion-positive. Multiple MRI sequences show a large mass centered in the left temporal lobe (arrows), with partial effacement of the left lateral ventricle (arrowhead). The predominantly solid mass shows restricted diffusion (**A,B**) and multiple foci of susceptibility (**C**), which may be related to calcification or prior hemorrhage. The solid component is T2 isointense to the cortex (**D**) and shows heterogeneous enhancement (**E**). Case courtesy of Dr. Aashim Bhatia, Children's Hospital of Philadelphia.

On imaging, these tumors are mixed solid and cystic, with the solid component demonstrating similar T2 signal intensity to that of the cortex (Figure 2) (9). These tumors often involve the lateral ventricles and are intraventricular and/or paraventricular. Vasogenic edema surrounding the tumor is variable.

Magnetic resonance (MR) spectroscopy of supratentorial ependymomas shows elevated choline and reduced N-acetylaspartate, similar to those of other brain tumors. This technique is most useful when distinguishing between tumor recurrence and post-treatment effects or radiation necrosis, rather than differentiating between ependymomas and other tumors or subtypes of ependymomas (10).

Differential considerations for supratentorial ependymomas are broad, and imaging cannot provide a definitive diagnosis. For parenchymal tumors, differential considerations include diffuse high-grade pediatric gliomas, all types except diffuse midline gliomas, H3 K27- altered, embryonal tumors such as atypical teratoid/rhabdoid tumors or embryonal tumors with multilayered rosettes, and circumscribed astrocytic gliomas, such as astroblastomas (2). These tumors may all present as supratentorial, solid and cystic, heterogeneously enhancing masses. If the mass is intraventricular, the differential diagnosis includes central neurocytoma, choroid plexus tumor, and subependymal giant cell astrocytoma. Central neurocytomas are generally seen in patients who are in their third to fifth decade of life and have elevated glycine on MR spectroscopy (11). Lateral ventricle choroid plexus tumors may show an enlarged choroidal artery feeding the tumor on CT or conventional angiography (11). Subependymal giant cell tumors are seen in tuberous sclerosis and usually arise near the foramen of Monro (11). Ultimately, tissue sampling is necessary to distinguish between supratentorial ependymomas and other neoplasms.

## Posterior fossa ependymomas

Posterior fossa ependymomas often present with signs and symptoms of increased intracranial pressure and ataxia. Molecular markers are divided into PFA and PFB based on methylation.

**Figure 3:**
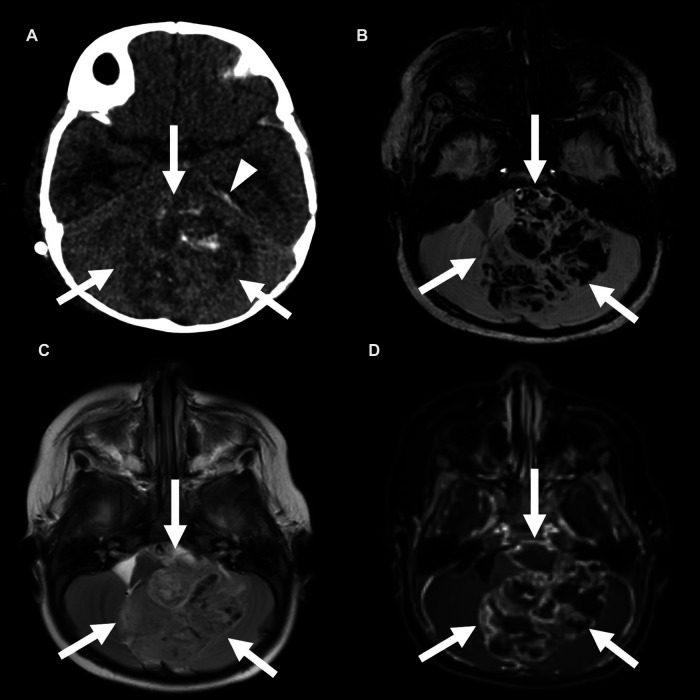
Posterior fossa (PF) group A ependymoma in a 3-year-old girl. Non-contrast CT (**A**) and axial SWI MRI images (**B**) show a large, hemorrhagic mass in the PF that effaces the fourth ventricle. A small amount of subdural blood products is also seen along the left tentorium (arrowhead). This mass is similar in T2 signal compared with the adjacent brain parenchyma (**C**) and shows heterogeneous enhancement (**D**).

Posterior fossa group A tumors frequently occur in infants and young children, with a median age of 3 years and a slight prevalence in male patients. The prognosis of PFA ependymomas is worse compared with that of PFB ependymomas, with an increased frequency of recurrence and metastasis (6, 12). The overall 10-year survival rate is 56%. These lesions tend to involve the roof or lateral aspects of the fourth ventricle with extension into the foramen of Luschka (Figure 3) (12). These tumors are less likely to enhance compared to PFB tumors but are more likely to contain calcifications than PFB tumors (13). There is no difference between PFA and PFB lesions in terms of the likelihood of extending beyond the fourth ventricle (13).

**Figure 4:**
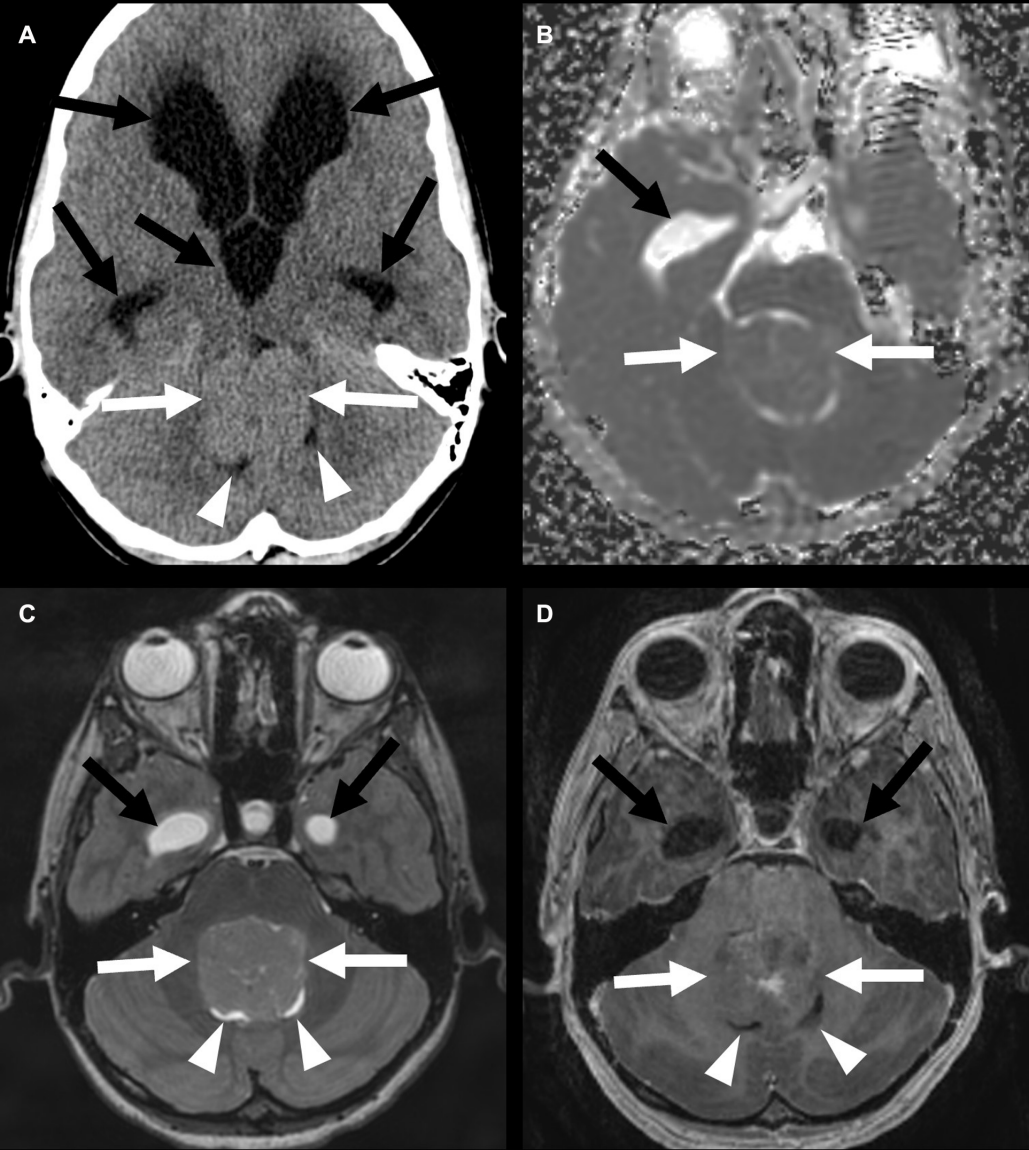
Posterior fossa (PF) group B ependymoma in a 12-year-old boy (white arrows). Non-contrast head CT (**A**) shows a mass in the PF without calcification. Apparent diffusion coefficient map shows no restricted diffusion (**B**). The mass has a similar T2 signal as the adjacent cortex (**C**) and shows heterogeneous enhancement (**D**). There is an associated almost complete effacement of the fourth ventricle (arrowheads) and supratentorial hydrocephalus (black arrows).

Posterior fossa group B tumors occur predominantly in adolescents and young adults, with a median age of 30 years, and are slightly more common in females. There is a better prognosis with a 10-year survival rate of 88% (6, 12). These lesions tend to arise from the floor of the fourth ventricle in the midline (Figure 4) (12). These tumors are also more likely to have cystic components and demonstrate enhancement of the solid components than PFA tumors. Calcifications are less common in PFB tumors than in PFA tumors (13).

Magnetic resonance spectroscopy of PF ependymomas also shows elevated choline and reduced N-acetylaspartate. Increased myoinositol is suggestive of PF ependymoma rather than medulloblastoma or hemangioblastoma (14).

In PF ependymomas, the main differential consideration is medulloblastoma, which is the most common PF tumor in this age group (15). Medulloblastomas always show restricted diffusion, which is not seen in low-grade ependymomas, although diffusion restriction may be difficult to interpret in masses with calcification or hemorrhage (15). Pilocytic astrocytomas are the second most common PF tumor in children but are more likely to have a large cystic component, which is less typical of ependymomas (15). Ependymomas are more likely than these tumors to extend through the foramen of Luschka or the foramen of Magendie (15). Differential considerations for intraventricular masses also include subependymoma and choroid plexus tumors. Subependymomas, as discussed in greater detail below, are more common in adults and are classically found in the inferior aspect of the fourth ventricle (11). Choroid plexus tumors are difficult to distinguish from other tumors based on imaging but may have long vascular pedicles and lobulated contours (11). As with supratentorial ependymomas, tissue sampling is necessary for definitive diagnosis.

## Spinal ependymomas

Spinal ependymomas are the second most common intramedullary neoplasm in children and are the most common intramedullary neoplasm in adults (16). These tumors comprise 30% of pediatric intramedullary tumors. Patients with neurofibromatosis type 2 are at an increased risk of developing these tumors. The most common symptoms at presentation are sensory changes and pain, with some patients also experiencing weakness or incontinence (17). Sensory symptoms are more common, possibly due to the central location of these tumors.

**Figure 5:**
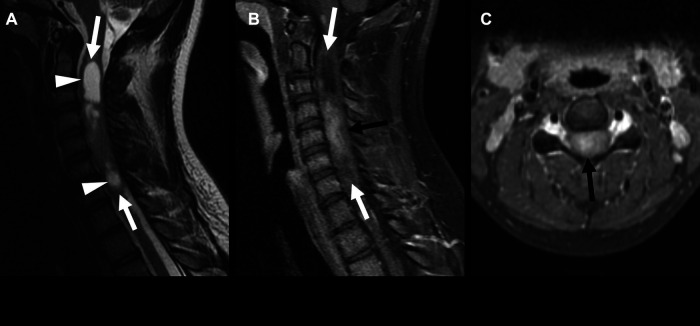
Spinal ependymoma, not elsewhere classified, in a 17-year-old girl with a history of neurofibromatosis 2, in the cervical spinal cord (arrows). Sagittal T2-weighted images show cystic areas (**A**) (arrowheads), and sagittal and axial postcontrast images show enhancement of the solid components of the tumor (**B, C**) (black arrows). There is an associated expansion of the spinal cord and effacement of the extra-axial spaces of the mid to distal cervical spine.

While ependymomas can occur at any level of the spinal cord, they are more common in the cervical cord (17). These tumors are T1 iso- to hypointense and demonstrate heterogeneous enhancement (Figure 5). Perilesional edema and cystic foci are common. Spinal ependymomas are less frequently calcified and may present with the “cap sign,” with peripheral T2 hypointensity due to hemosiderin deposition.

A small group of spinal ependymomas is defined by MYCN amplification. This tumor type is rare, with fewer than 30 cases reported in the literature but appears to be more common in adolescent and young adult females (18). Spinal ependymomas associated with MYCN amplification are more aggressive, with an increased likelihood of recurrence and metastasis, and decreased progression-free and overall survival (18). These tumors would generally have been classified as grade 3 anaplastic ependymoma in the previous WHO classification.

**Figure 6:**
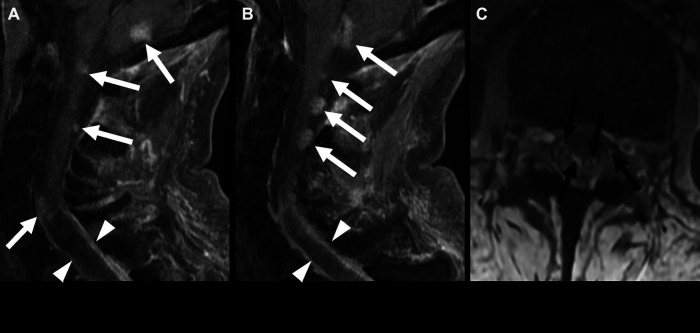
Spinal ependymoma, MYCN amplified, in a 46-year-old woman. Sagittal post-contrast MRI (**A,B**) shows multiple enhancing nodules in the visualized posterior fossa and cervical spinal cord (white arrows). A partially cystic lesion extends from the mid-cervical spine into the thoracic spine (arrowheads). Multiple enhancing nodules in the cauda equina are consistent with leptomeningeal spread (**C**) (black arrows).

MYCN-amplified tumors are typically large and involve multiple vertebral levels. They may be intramedullary or extramedullary and often have leptomeningeal metastases at presentation (Figure 6) (19). Tumors without MYCN amplification are smaller and less aggressive. MR spectroscopy is generally not performed in spinal tumors because of the small axial cross-sectional tumor size and the susceptibility artifact related to the adjacent bone (10).

The main differential consideration is a spinal astrocytoma, which is the most common spinal cord tumor in children (16). Spinal astrocytomas tend to be less well-defined and involve a larger segment of the cord, although pathology is required for a definitive diagnosis. Other less common differential considerations include cavernous malformations or diffuse midline gliomas. Cavernous malformations of the spinal cord present as multilobulated lesions with heterogeneous signals (popcorn appearance) and multiple foci of susceptiblity, similar to their brain parenchymal counterparts (20). Diffuse midline gliomas are classically found in the brain stem but can be found in any midline CNS structure, including the spinal cord (21).

## Myxopapillary ependymomas

The classification of myxopapillary ependymoma remains unchanged in the WHO 2021 classification, as molecular classification does not provide additional clinical utility. There is, however, an increase in grade from grade 1 to grade 2 despite their slow growth rate, given that the rate of local recurrence is similar to that of other spinal ependymomas (4).

These tumors are the most common tumors of the conus medullaris and filum terminale. In the pediatric population, they are more prone to dissemination in cerebrospinal fluid (CSF) than those in adults (22). Patients frequently present with low back, leg, or sacral pain, and some may present with symptoms of cauda equina compression such as lower extremity weakness or sphincter dysfunction.

**Figure 7:**
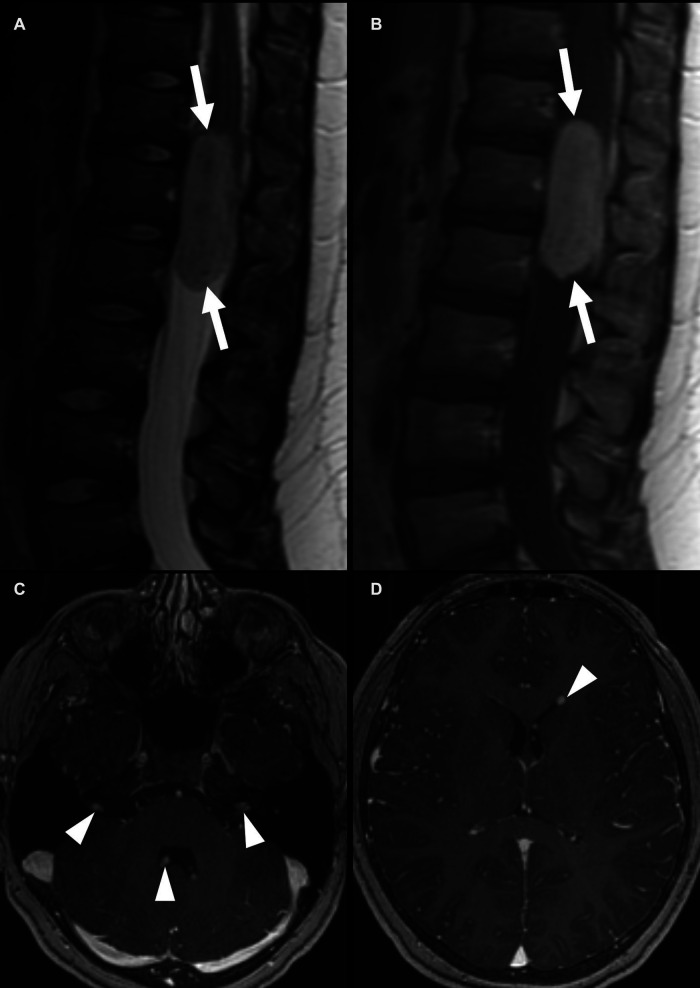
Myxopapillary ependymoma in a 9-year-old boy. Centered at the cauda equina cauda equina, there is an intradural, extramedullary mass that is T2 intermediate (**A**), with homogeneous enhancement (**B**) (white arrows). This mass was treated with resection, with subsequent intracranial spread, with enhancing nodules in the fourth ventricle and bilateral internal auditory canals (**C**) and at the left frontal horn (**D**) (arrowheads).

On imaging, myxopapillary ependymomas are intradural, circumscribed, oval or sausage-shaped masses located at the cauda equina (Figure 7). They may be associated with hemorrhage with calcification or cystic degeneration. There is displacement and possible encasement or compression of the cauda equina nerve roots (17). These tumors tend to be T1 isointense and T2 hyperintense compared to those in the spinal cord. Hemorrhage may cause areas of T2 hypointensity. Generally, homogeneous contrast enhancement is noted, but heterogeneous enhancement may be seen with hemorrhage. Magnetic resonance spectroscopy is generally not performed for spinal tumors because of the small axial cross-sectional tumor size and the susceptibility artifact related to the adjacent bone (10).

Differential considerations include peripheral nerve sheath tumors such as schwannoma or paraganglioma, as well as metastatic disease. Schwannomas are the second most common tumor of the cauda equina, accounting for approximately one-third of spinal nerve root tumors (23). Most of these tumors are sporadic but may be associated with neurofibromatosis type 2. Paragangliomas are uncommon and are highly vascular lesions associated with prominent flow voids (17, 23). Metastatic disease may be considered if there is a known primary malignancy, either as a drop metastasis from an intracranial primary or as a distant metastasis from a primary malignancy elsewhere in the body.

## Subependymomas

There is no change in the WHO classification for subependymomas, which continue to be classified based on morphologic criteria. No clinical utility is found for molecular analysis at this time, and these tumors continue to be categorized as WHO grade 1 (4). Subependymomas are uncommon tumors that typically present in middle-aged patients, usually in their fifth or sixth decade of life, and are more common in men (24). These tumors are often found incidentally, although large tumors may cause signs and symptoms related to mass effect or CSF obstruction.

**Figure 8:**
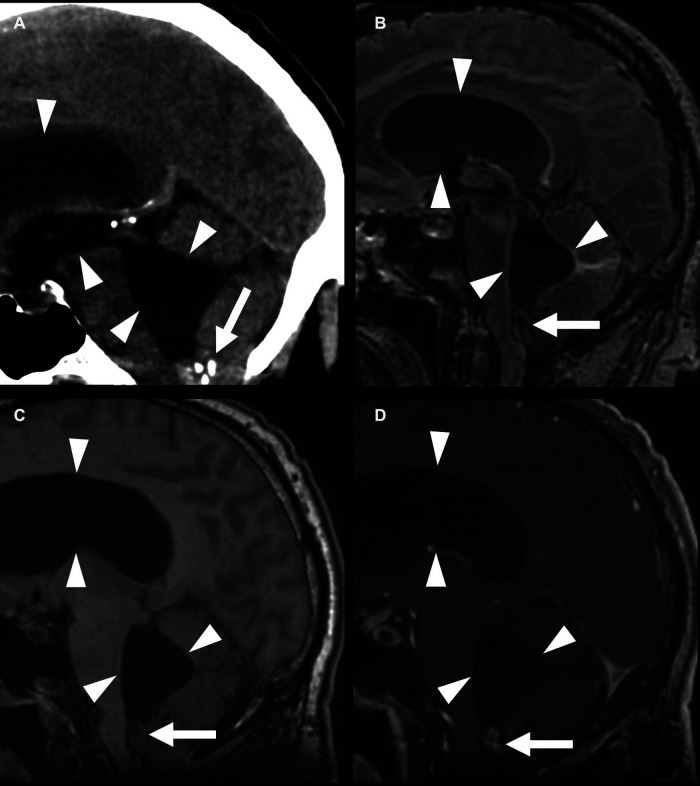
Fourth-ventricle subependymoma in a 54-year-old man. CT shows a partially calcified mass at the inferior aspect of the fourth ventricle (**A**) (arrows), with an associated supratentorial and infratentorial hydrocephalus (arrowheads). This mass is FLAIR (**B**) and T1 (**C**) isointense, with post-contrast enhancement (**D**). The central T1 and FLAIR hypointensity is due to susceptibility due to calcifications.

**Figure 9:**
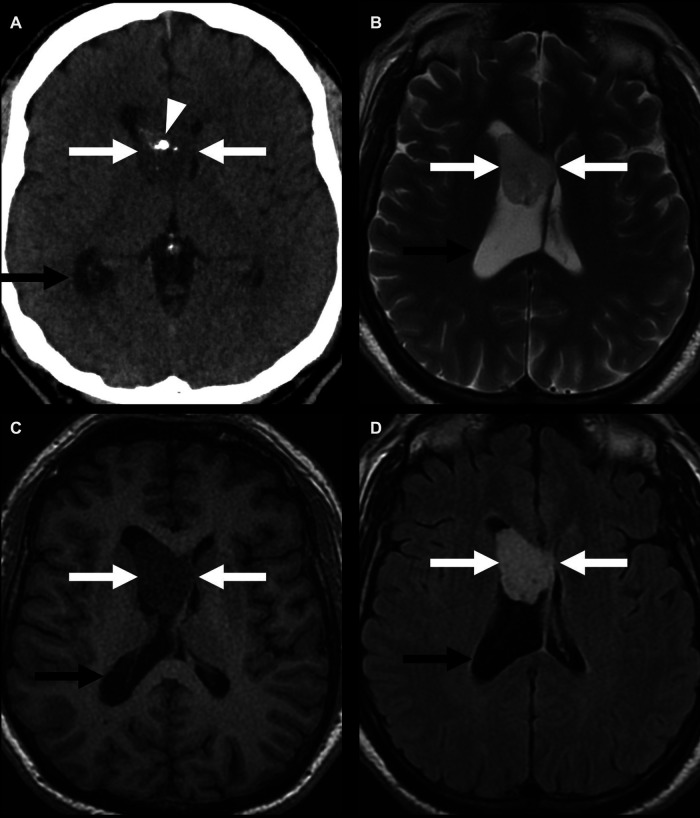
Lateral-ventricular subependymoma in a 63-year-old man (arrows). CT shows a mass in the frontal horn of the right lateral ventricle containing calcifications (arrowhead) (**A**). This mass is mildly T2 hyperintense (**B**) and T1 hypointense (**C**) and shows homogeneous post-contrast enhancement (**D**). The associated obstructive hydrocephalus of the right lateral ventricle (black arrow) is due to blockage of the foramen of Monro.

Subependymomas are homogeneous when small and may be heterogeneous and contain calcifications and cystic foci when larger (Figures 8, 9). These tumors are usually hypodense on CT. On MRI, they are T1 iso- or hypointense and T2/FLAIR hyperintense, compared with adjacent parenchyma, and typically show little or no enhancement (Figure 7) (24). Most of these tumors occur in the inferior aspect of the fourth ventricle but may also be found in the other ventricles or in the central canal of the spinal cord (11). MR spectroscopy of supratentorial subependymomas shows decreased N-acetylaspartate relative to normal control, without elevated choline (25).

Small tumors in the classic location with consistent imaging features and no associated hydrocephalus are presumed to be subependymomas, and most of these tumors are not sampled or removed. Otherwise, tissue sampling is needed for a definitive diagnosis. Differential considerations include other intraventricular neoplasms such as ependymoma and choroid plexus papilloma. In the lateral ventricles, central neurocytoma or subependymal giant cell astrocytoma may also be considered. Central neurocytomas show an elevated choline/creatine ratio on MR spectroscopy compared to subependymomas (25). Subependymal giant cell astrocytomas are almost always seen in tuberous sclerosis.

## Conclusion

Clinical presentation and prognosis of ependymal tumors vary depending on tumor location and type. The WHO 2021 classification incorporates more up-to-date information on tumor genomics. Given the different prognoses conferred by the molecular profile of the tumor, it is important for radiologists to understand these classifications to provide more accurate and useful reports. Accurate use of these updated classifications will also provide a basis for research.

As more information about the molecular profiles of tumors becomes available, more molecular tumor types will be defined, and the classification systems will be updated to incorporate new information. However, the fundamental information provided by imaging, such as tumor localization, assessment of disease extent, and effect on surrounding structures, will remain unchanged and central to the practice of radiology.
